# Continuous exposure of isoprenaline inhibits myoblast differentiation and fusion through PKA/ERK1/2-FOXO1 signaling pathway

**DOI:** 10.1186/s13287-019-1160-x

**Published:** 2019-02-28

**Authors:** Shao-juan Chen, Jing Yue, Jing-Xuan Zhang, Miao Jiang, Tu-qiang Hu, Wei-dong Leng, Li Xiang, Xin-yuan Li, Lei Zhang, Fei Zheng, Ye Yuan, Lin-yun Guo, Ya-mu Pan, Yu-wen Yan, Jia-ning Wang, Shi-You Chen, Jun-ming Tang

**Affiliations:** 1 Department of Cardiology, and Institute of Clinical Medicine, Renmin Hospital, Hubei University of Medicine, Shiyan, 442000 Hubei People’s Republic of China; 2 Department of Stomatology, Taihe Hospital, Hubei University of Medicine, Shiyan, 442000 Hubei People’s Republic of China; 3 Department of Physiology, School of Basic Medical Sciences, Hubei University of Medicine, Shiyan, 442000 Hubei People’s Republic of China; 4 Department of Stomatology, Renmin Hospital, Hubei University of Medicine, Shiyan, 442000 Hubei People’s Republic of China; 5 Institute of biomedicine and Key Lab of Human Embryonic Stem Cell of Hubei Province, Hubei University of Medicine, Shiyan, 442000 Hubei People’s Republic of China; 6 Department of Physiology & Pharmacology, The University of Georgia, Athens, GA30602 USA

**Keywords:** Isoprenaline, Adrenergic receptor, Differentiation, Myoblast fusion, PKA, ERK1/2, FOXO1

## Abstract

**Aim:**

The objective of this study is to determine if exuberant sympathetic nerve activity is involved in muscle satellite cell differentiation and myoblast fusion.

**Methods and results:**

By using immunoassaying and western blot analyses, we found that β1 and β2-adrenergic receptors (AdR) were expressed in C2C12 cells. The differentiated satellite cells exhibited an increased expression of β2-AdR, as compared with the proliferating cells. Continuous exposure of isoprenaline (ISO), a β-AdR agonist, delayed C2C12 cell differentiation, and myoblast fusion in time- and dose-dependent manner. ISO also increased short myotube numbers while decreasing long myotube numbers, consistent with the greater reduction in MyHC1, MyHC2a, and MyHC2x expression. Moreover, continuous exposure of ISO gradually decreased the ratio of PKA RI/RII, and PKA RI activator efficiently reversed the ISO effect on C2C12 cell differentiation and myoblast fusion while PKA inhibitor H-89 deteriorated the effects. Continuous single-dose ISO increased β1-AdR expression in C2C12 cells. More importantly, the cells showed enhanced *phospho-*ERK1/2 levels, resulting in increasing *phospho-*β2-AdR levels while decreasing β2-AdR levels, and the specific effects could be abolished by ERK1/2 inhibitor. Furthermore, continuous exposure of ISO induced FOXO1 nuclear translocation and increased the levels of FOXO1 in nuclear extracts while reducing pAKT, p-p38MAPK, and pFOXO1 levels. Conversely, blockade of ERK1/2 signaling partially abrogated ISO effects on AKT, p38MAPK, and FOXO1signaling, which partially restored C2C12 cell differentiation and myoblast fusion, leading to an increase in the numbers of medium myotube along with the increased expression of MyHC1 and MyHC2a.

**Conclusion:**

Continuous exposure of ISO impedes satellite cell differentiation and myoblast fusion, at least in part, through PKA-ERK1/2-FOXO1 signaling pathways, which were associated with the reduced β2-AdR and increased β1-AdR levels.

**Electronic supplementary material:**

The online version of this article (10.1186/s13287-019-1160-x) contains supplementary material, which is available to authorized users.

## Introduction

Muscular dystrophy (MD) is a destructive neuromuscular disease characterized by progressive muscle weakness, muscle atrophy, and cardiac dysfunction [1]. In addition to the primary muscular defects, another possible contributor to the generation of pathology in MD is the autonomic dysfunction [2]. Indeed, an autonomic imbalance has been observed in which sympathetic activity is increased while coupled with diminished parasympathetic activity, contributing to the development of dilated cardiomyopathy (DCM), ventricular arrhythmias, and sudden cardiac death in patients with Duchenne and Becker MD [1, 3]. In response to norepinephrine (NE) and epinephrine (E) released by components of the sympathetic nerve system (SNS), activation of β1- and β2-adrenergic receptors (AdRs), as a result of NE and E binding to specific AdRs, is the most important physiologic mechanism to acutely increase cardiac performance via positive inotropic, dromotropic, and chronotropic effects [4, 5]. However, most studies have focused on the impact of autonomic dysfunction on cardiac performance and morphology in MD with little attention to its possible role in the development of skeletal myopathy.

Accumulated data have shown that activated β-AdR, a typical indication of excessive SNS, is involved in the development of essential hypertension and heart failure (HF) [4–6]. Indeed, the over-activated SNS exacerbates the HF with clinical features such as physical activity limitation, fatigue, and skeletal muscle atrophy [7]. It has been speculated that autonomic imbalance inhibits the anabolism of skeletal muscle, attributing to the reduced expression and sensitivity of β2-AdR in skeletal muscle [8]. These results suggest that β-AdR is involved in MD.

Muscle-resident satellite cells contribute to muscle renewal and maintenance in accordance with physical demand and repair after disease or injury [9]. The regenerative capacity of satellite cells in myotonic dystrophy is constitutively impaired, causing loss of muscle fibers with disease progression and aging [10, 11]. Based on the involvement of autonomic nervous dysfunction in skeletal muscle atrophy, we hypothesize that the changes of β-AdR activities impair satellite cell differentiation and myoblast fusion, resulting in skeletal muscle atrophy. In this study, we used β-AdR agonist isoproterenol (ISO) to stimulate an abnormal activation of β1-AdR and inactivation of β2-AdR to uncover the possible relationship between satellite cells and muscle atrophy due to the over-activation of SNS.

## Method

### Reagents and chemicals

CPG 20712A was purchased from Tocris Bioscience. H 89 (S1582) was purchased from Selleckchem. Isoprenaline (ISO, I5627), N6-Benzoyladenosine-3′,5′-cyclic monophosphate sodium salt (N^6^-Bz-cAMP, B4560), and PD98059 (P215) were purchased from Sigma-Aldrich and reconstituted in their corresponding vehicle according to the manufacturer’s instructions.

### The culture and differentiation of C2C12 cells

The myoblast C2C12 cells that were purchased from Cell bank of the Chinese Academy of Sciences were inoculated in 75-cm^2^ culture dish and cultured with high glucose DMEM containing 10% fetal bovine serum (FBS) at 37 C and 5% CO_2_. When cells confluence reached 70% to 80%, the culture medium was replaced with high glucose DMEM containing 2% horse serum (HS) to induce C2C12 cell differentiation. The C2C12 cell differentiation into myotubes was observed every day under a microscope. The formation of myotubes from C2C12 cells were detected by myotube markers at the 3rd, 5th, and 8th day of differentiation [12].

### ISO delivery method in vitro

C2C12 cells cultured in high glucose DMEM containing 2% HS at 70% confluence were exposed to ISO transiently or continuously. In brief, the transient ISO-delivery is to add ISO for only one time on the first day of the differentiation and then replace with fresh differentiation medium every day. The continuous exposure is to add the same dose of ISO each day when the differentiation medium is replaced. Different dosages of ISO (i.e., 10^−8^ M, 10^−7^ M, 10^−6^ M, or 10^−5^ M) were also applied in the subsequent experiments. Myotube formation was analyzed 5–8 days after the ISO treatment.

### Adrenergic receptor assay

C2C12 cells were inoculated to 24-well plates with 1.25 × 10^4^cells/well. The cells were cultured irregular medium for 24 h or in the differentiation medium for 6 days. Cells were then fixed with 4% polyoxymethylene for 15 min and washed with PBS for 3 times (5 min/time). Five percent goat serum and 0.3% Triton-100 were used to block the nonspecific antigen for 30 min. Subsequently, the primary antibodies including β1 (ab3442)- and β2 (ab36956)-adrenergic receptor (AdR, 1:100, Abcam) were added onto the corresponding wells and incubated overnight at 4 °C. After washing with PBS 3 times for 15 min, cells were incubated with corresponding fluorescent dye-labeled secondary antibodies (1:250) at room temperature for 2 h. Cells were then observed and photographed under fluorescence microscope [13]. To quantitatively analyze the expression of adrenergic receptors in C2C12 and differentiated cells, cell lysates were collected for western blot analyses 6 days after the culture or differentiation.

### Immunofluorescence staining

C2C12 cells were treated similarly as in AdR assay above. Primary antibodies against MyHC (sc-20641, 1:150, Santa Cruze) and FOXO1 (SC-9808, 1:150, Santa Cruze) were added into the corresponding wells and incubated overnight at 4 °C. After washing with PBS 3 times for 15 min, cells were incubated with corresponding fluorescent dye-labeled secondary antibodies (1:250) at room temperature for 2 h. DAPI (Molecular Probes) was used to stain nuclei. The corresponding images were observed and photographed under a fluorescence microscope [14].

### Myotube morphology

Cells were stained for MyHC using MyHC polyclonal rabbit anti-mice antibody (sc-20,641, 1:150, Santa Cruze) followed by anti-rabbit TRITC or FITC-labeled secondary antibody (Jackson Lab, 1:400, USA). DAPI was used to stain nuclei. Myotube was defined as 3+ nucleuses within a cellular structure in order to rule out myoblast cells undergoing mitosis. The images of five locations including up, down, left, right sides, and center of each slide were photographed using an 80i Nikon fluorescent microscope (Nikon, Japan). A total of 30 images/group within six repeats were taken by using the same imaging parameters. Images were analyzed by two pathologists using Image J (Java) software (National Institutes of Health, USA) in a double-blind manner. Morphology was assessed by myotube length, number of myotube per view, and number of myoblast fusion (nuclei numbers as the indicator) per myotube [15–17]. In order to facilitate the description of myotube characteristics, the myotubes were divided into 3 types including short, medium, and long myotubes. Short myotube was defined as those shorter than 200 μm with less than 5 myoblast fusions, and long myotubes were defined as those longer than 400 μm with more than 10 myoblast fusion; medium myotube were those between the short and long myotubes.

### Quantitative RT-PCR

Total RNA was extracted from C2C12 cells using TRIzol reagents (Life Technologies) and transcribed into cDNA using the SuperScript II cDNA kit (Life Technologies). Quantitative PCR was performed using SYBR green PCR master mix (Applied Biosystems) in RotorGene 6000 Real-Time PCR System (Qiagen, Mannheim, Germany). The transcript levels of interested genes in the corresponding groups were compared after normalization to GAPDH levels [18]. The primers used are shown in Table 1.

**Table 1:** The sequences of primers of qPCR

Gene	Forward	Reverse
MyoG	5’-GAGACATCCCCCTATTTCTACCA-3’	5’-GCTCAGTCCGCTCATAGCC-3’
MyoD1	5’-CCACTCCGGGACATAGACTTG-3’	5’-AAAAGCGCAGGTCTGGTGAG-3’
MyHC1	5’-CAAGCAGCAGTTGGATGAGCGACT-3’	5’-TCCTCCAGCTCCTCGATGCGT-3’
MyHC2a	5’-AGAGGACGACTGCAGACCGAAT-3’	5’-GAGTGAATGCTTGCTTCCCCCTTG-3’
MyHC2b	5’-ACGCTTGCACACAGAGTCAG-3’	5’-CTTGGACTCTTCCTCTAGCTGCC-3’
MyHC2x	5’-ACCAAGGAGGAGGAACAGCAGC-3’	5’-GAATGCCTGTTTGCCCCTGGAG-3’
GAPDH	5’-ATGACTCCACTCACGGCAAA-3’	5’-ATGATGACCCTTTTGGCTCC-3’

qPCRs were performed to identify the traits of satellite cell differentiation and muscle fibers by using the specific primers of satellite cell differentiation markers including MyoD and MyoG, type I muscle fiber makers like MyHC1, and type II muscle fiber makers such as MyHC2a, MyHC2b, and MyHC2X. GAPDH as an internal control

### Western blot

C2C12 cells were homogenized on ice in 0.1% Tween-20 homogenization buffer containing protease inhibitors. Nuclear and cytosolic proteins in C2C12 cells were isolated and collected using NE-PER Nuclear and Cytoplasmic Extraction Reagents by following the manufacturer’s instruction (78,835, Thermo Scientific, USA). 20 μg of proteins were resolved in 7 or 10% SDS-PAGE gel and transferred onto a polyvinylidene fluoride (PVDF) membrane (Millipore). After being blocked with 5% nonfat milk, the membrane was incubated with primary antibody (1:1000 dilution) for 90 min followed by incubation with horseradish peroxidase (HRP)-conjugated secondary antibodies (anti-rabbit IgG, anti-goat IgG, 1:10000; Santa Cruz). Protein expression was visualized by enhanced chemiluminescence reaction and measured by densitometry. The primary antibody used were as follows: α-tubulin (T9026, 1:5000, Sigma), β1-AdR (ab3442, 1:1000, Abcam), β2-AdR (ab182136, 1:1000, Abcam), p-β2-AdR (PA5-36784, 1:500, Thermo), PKAαcat (sc-28315, 1:500, Santa Cruze), PKAγ cat (sc-514,087, 1:500, Santa Cruze), PKAα/β/ϒcat (sc-365615, 1:500, Santa Cruze), PKA RIα (sc-136231, 1:500, Santa Cruze), PKA Riβ (sc-100414, 1:500, Santa Cruze), PKA RIIα (sc-136262, 1:500, Santa Cruze), PKA RIIβ (sc-376778, 1:500, Santa Cruze), AKT (#9272 s, 1:500, Cell Signaling), *p*AKT (#4058 s, 1:500, Cell Signaling), extracellular signal-regulated kinase (ERK1/2, SC-94, SC-154, 1:500, Cell Signaling), *p*ERK1/2(4370 s, 1:500, Cell Signaling), mitogen-activated protein kinase (*p*38MAPK, #9211, 1:500, Cell Signaling), *p*-p38MAPK (sc-7973, 1:500, Cell Signaling), PKCα (sc-8393,1:500,Santa Cruze), *p*PKCα (sc-12356, 1:500, Santa Cruze), FOXO1 (sc-9808, 1:500, Santa Cruze), *p*FOXO1(sc-101681, 1:150, Santa Cruze), Histone H3(ab6002, 1:500, ABCAM), MuRF1 (ab77577, 1:500, Abcam), MyHC (sc-20641, sc-376157, 1:500, Santa Cruze), and MyoG (sc-12732, 1:500, Santa Cruze), respectively. The semi-quantitative analysis was done by using the Image J software to measure the gray value [19].

### Statistical analysis

Data shown are mean ± SD. Statistical significance between two groups was determined by paired or unpaired Student’s *t* test. Results for more than two experimental groups were evaluated by one-way ANOVA to specify differences between groups. *P* < 0.05 was considered significant.

## Results

### Continuous stimulation with ISO inhibited C2C12 cell differentiation and myotube formation

To explore the potential role of ISO in C2C12 cell differentiation, myotube formation following C2C12 cell differentiation with 2% HS-DMEM was firstly observed under a microscope, and the differentiated C2C12 cells showed myotube formation characterized by multinucleated myoblast fusion in a time-dependent manner. During the first and second day of differentiation, C2C12 cells did not show obvious myotube formation. Starting from the third day, C2C12 cells began to form a few short myotubes. On the fourth day, the differentiated cells started to form long myotubes. The number of myoblast fusion in myotubes increased gradually and reached the peak on the 6th day (Additional file 1: Figure S1A). Meanwhile, the MyHC expression increased progressively and reached the peak at the 6th day as well (Additional file 1: Figure S1A-C).

To explore the effect of ISO on C2C12 cell differentiation, myotube morphology was observed by immunostaining of MyHC. As shown in Additional file 1: Figure S1D, continuous exposure of ISO to C2C12 cells shortened the MyHC-positive myotube and the myoblast fusion as compared with the transient exposure of ISO. Consistently, less reduction of MyHC protein level was detected by western blot in ISO-transiently treated cells compared to the ISO-continuously treated cells (Additional file 1: Figure S1D-F). These results suggested that the inhibition to C2C12 cell differentiation and myotube formation by continuous stimulation of ISO was stronger than the transient stimulation.

**Fig. 1:**
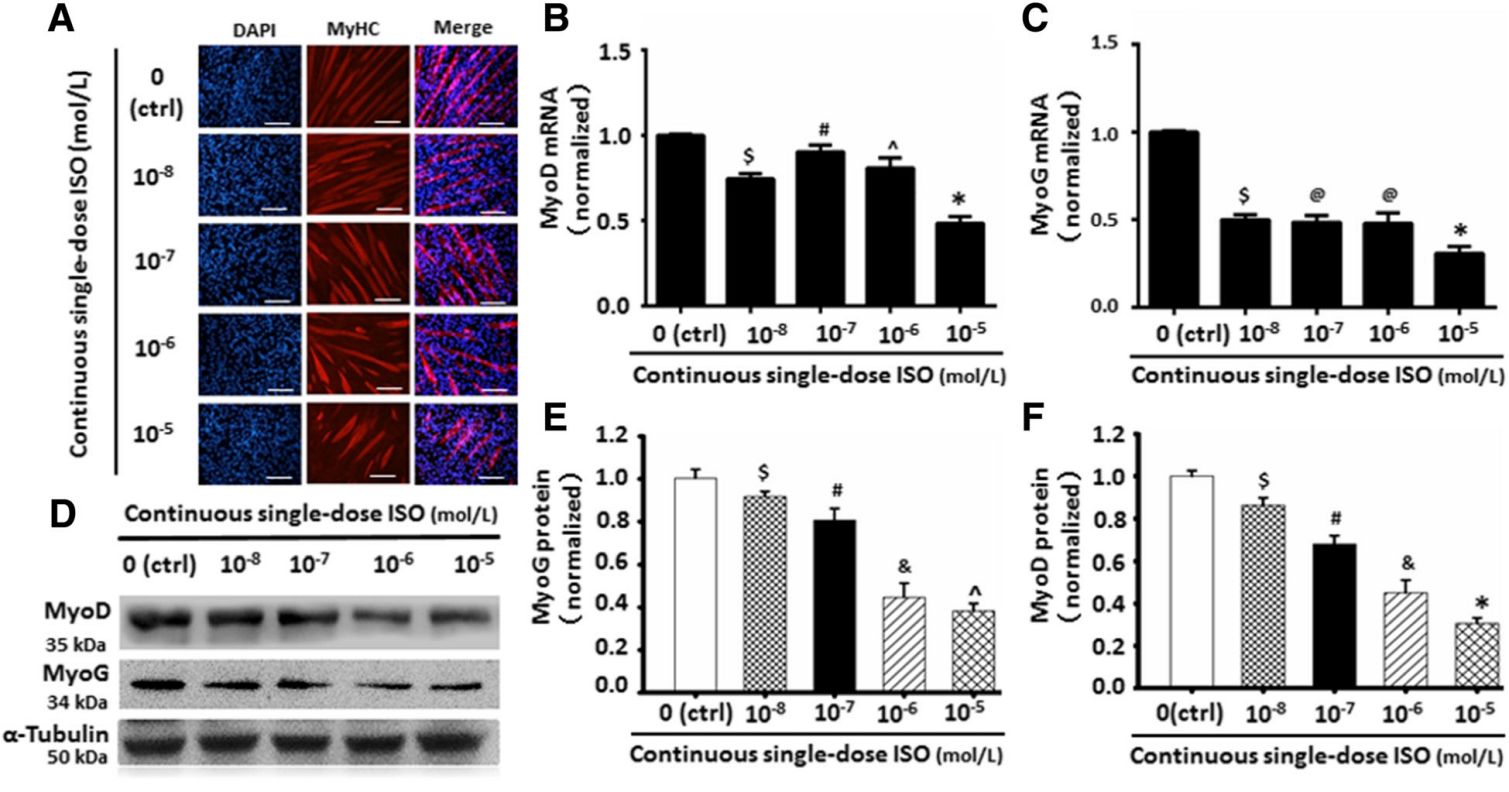
Continuous single-dose ISO dosage-dependently inhibited C2C12 cells differentiation and myoblast fusion. **a** The typical image of MyHC-positive muscle fibers in differentiated C2C12 cells exposed to different dose of ISO by using immunofluorescent staining. Red color indicates corresponding MyHC expression in the differentiated C2C12 cells; blue color indicates DAPI-labeled nuclei. **b**-**c** c The decreasing trend of C2C12 cells differentiation exposed to different dose of ISO were determined by Real-time PCR of satellite cell differentiation markers including MyoD and MyoG. ^$^*P* = 0.0051 vs. Ctrl; ^#^*P* = 0.0047 vs.10^-8^M ISO; ^*P* = 0.0263 vs. 10^-7^ M ISO; *P = 0.0033 vs. 10^-6^ M ISO; ^@^*P* = 0.0863 vs. 10^-8^ M ISO; *n* = 6. **d**-**f** Western blot were used to detect the above-mentioned protein levels to further confirm the traits of C2C12 cells differentiation inhibition following the continuous single-dose ISO stimulation. α-tubulin as the internal control. ^$^*P* = 0.0048 vs. Ctrl; ^#^*P* = 0.0039 vs.10^-8^ M ISO; ^&^*P* = 0.0054 vs. 10^-7^ M ISO; *=0.0196 vs. 10^-6^ M ISO; ^*P* = 0.0679 vs. 10^-6^ M ISO; *n* = 6

**Fig. 2:**
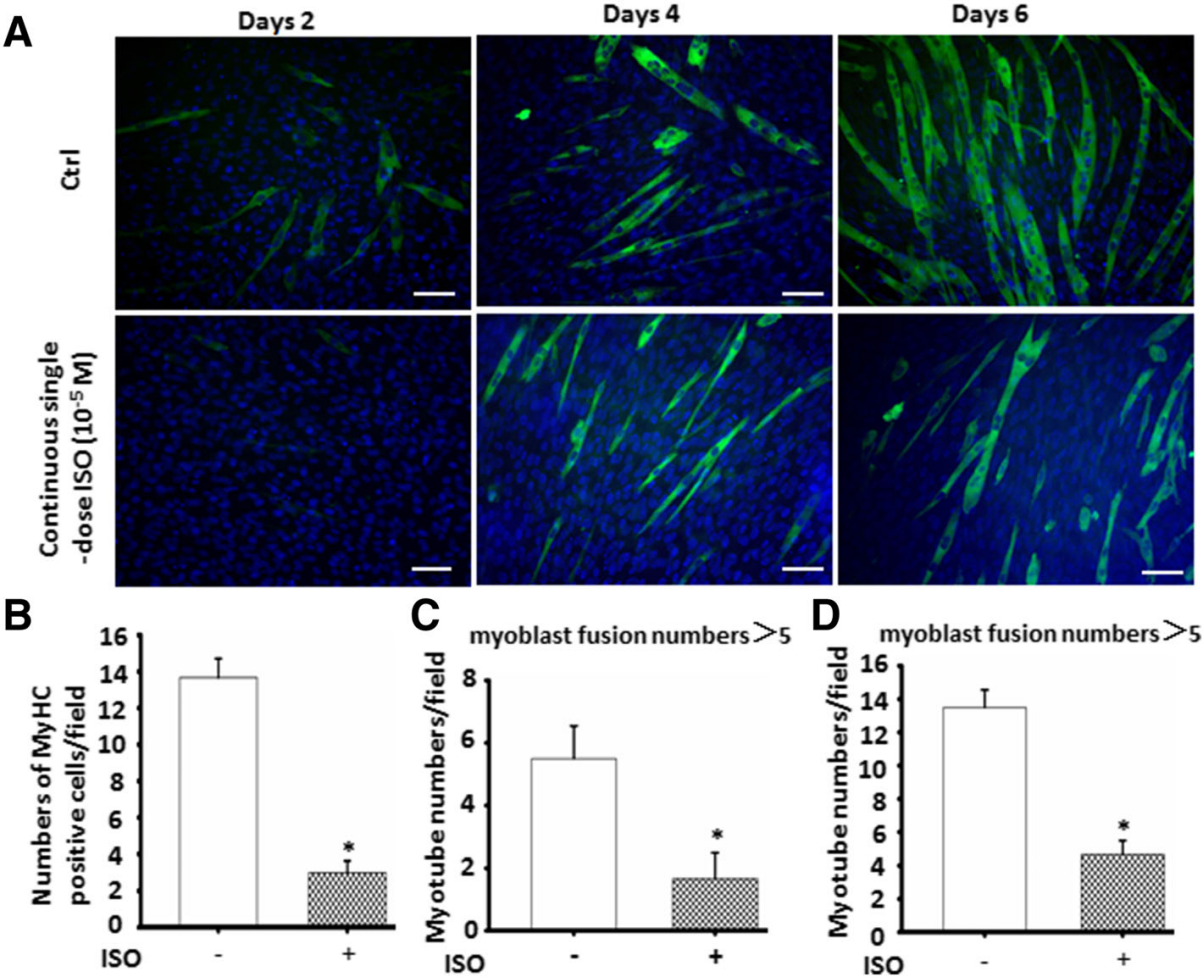
Continuous single-dose ISO time-dependently delayed C2C12 cells differentiation and myoblast fusion. **a** The typical image of myoblast fusion day 2, day 4 and day 6 after C2C12 cells differentiation with or without continuous single-dose ISO stimulation as determined by immunofluorescent staining of MyHC. Green color indicates MyHC; blue color indicates DAPI for nuclear labeling. **b** Continuous single-dose ISO prominently depressed the numbers of MyHC-positive cells day 2 after C2C12 cells differentiation. *P = 0.0000 vs. Ctrl. **c** Continuous single-dose ISO remarkably decreased the myotube numbers of more than 5 myoblast fusion day 4 after C2C12 cells differentiation. *P = 0.0000 vs. Ctrl. **d** Continuous single-dose ISO markedly reduced the myotube numbers of more than 5 myoblast fusion day 6 after C2C12 cells differentiation. *P = 0.0000 vs. Ctrl

To further observe the dose-dependent effect of ISO with continuous exposure on C2C12 cell differentiation and myoblast fusion, 10^−8^, 10^−7^, 10^−6^, or 10^−5^ mol/L of ISO was used to treat the cells. As shown in Fig. 1, the number and length of MyHC-positive myotubes were dosage-dependently reduced with continuous ISO stimulation. Moreover, continuous stimulation of ISO reduced the myoblast fusion competence (Additional file 1: Figure S2). In line with the myotube morphology, the mRNA and protein expression of satellite cell differentiation markers MyoD (Fig. 1b, d, f) and MyoG (Fig. 1c-e) were dose-dependently decreased while the myotube atrophy marker MuRF1 was induced (Additional file 1: Figure S3). Meanwhile, continuous single-dose ISO time-dependently delayed C2C12 cell differentiation and myoblast fusion (Fig. 2). These results further demonstrated that continuous single-dose ISO inhibited C2C12 cell differentiation and myotube formation.

### Continuous ISO stimulation altered the muscle fiber types

**Fig. 3:**
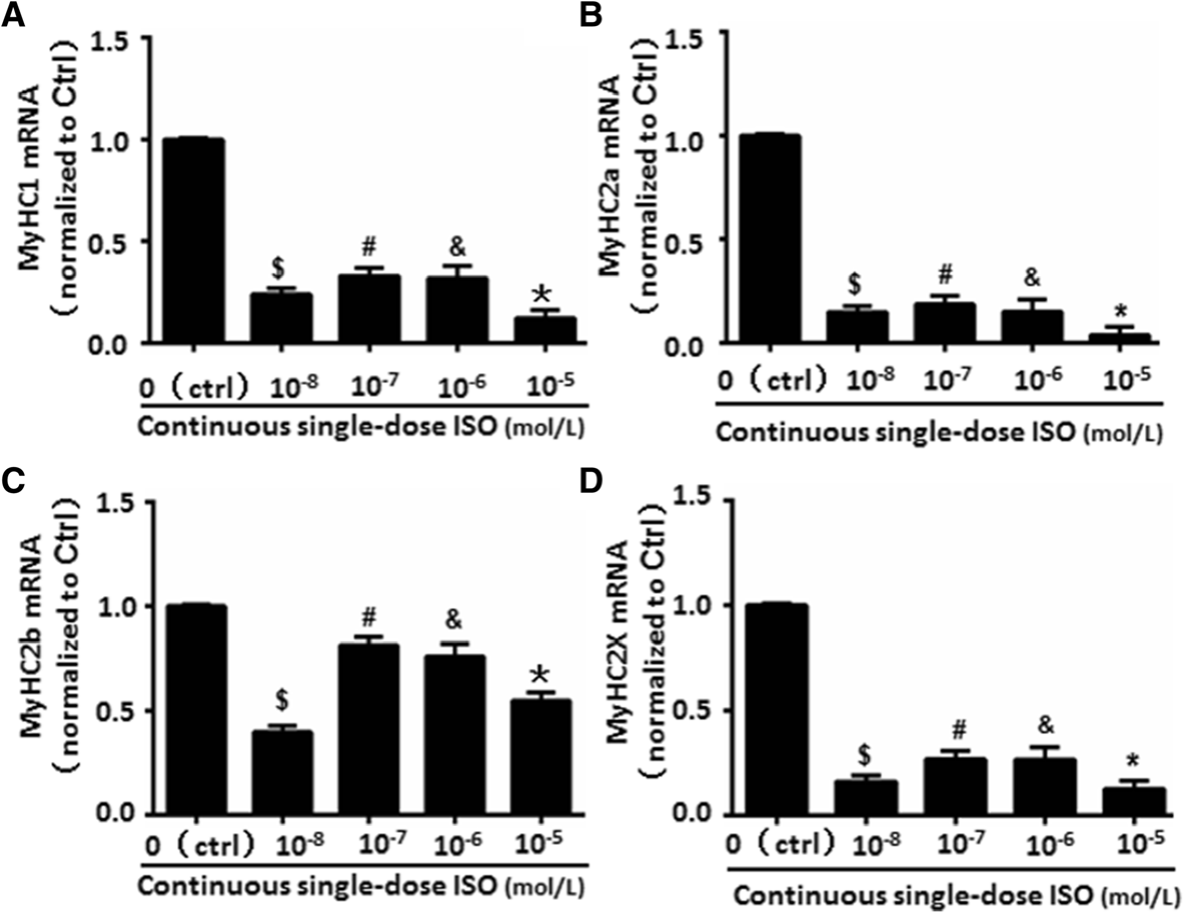
Continuous single-dose ISO altered the muscle fiber types. **a** MyHC1, as one of type I muscle fiber maker, were repressed in differentiated C2C12 cells continuously exposed to different doses of ISO by detecting the levels of mRNA using Real-time PCR. **b**-**d** Type II muscle fiber makers such as MyHC2a, MyHC2b and MyHC2x have shown the reduced changes of mRNA expressions in differentiated C2C12 cells following continuous single-dose ISO stimulation of mRNA expressions in differentiated C2C12 cells following continuous single-dose ISO stimulation. ^$^*P* = 0.0000 vs. Ctrl; ^#^*P* = 0.00368 vs. 10^-8^ M ISO; ^&^*P* = 0.0826 vs. 10^-7^ M ISO; *P = 0.0004 vs. 10^-6^ M ISO; *n* = 6

There are different types of muscle fibers formed by MyHC1, MyHC2a, MyHC2b, or MyHC2X. MyHC1-positive type I fiber shows a slim-long feature. MyHC2a, MyHC2b, and MyHC2X positive type II fiber has thick-short traits [20, 21]. In line with the reduced myotube formation following continuous ISO stimulation, MyHC1, MyHC2a, MyHC2b, and MyHC2X expression was markedly decreased (Fig. 3a–d). The reduction of MyHC1 (Fig. 3a), MyHC2a (Fig. 3b), and MyHC2X (Fig. 3d) was greater than MyHC2b (Fig. 3c). Although MyHC1, MyHC2a, and MyHC2b were dose-dependently decreased by ISO (Fig. 3a-c), the reduction for MyHC2X remained the same by 10^−8^~10^−5^ mol/L of ISO (Fig. 3d), suggesting a different effect of ISO on different MyHC isoforms. Nevertheless, these results suggested that continuous ISO stimulation inhibited the expressions of all MyHC isoforms.

### Continuous ISO stimulation delayed C2C12 cell differentiation and myoblast fusion through altering β-AdR activities

**Fig. 4:**
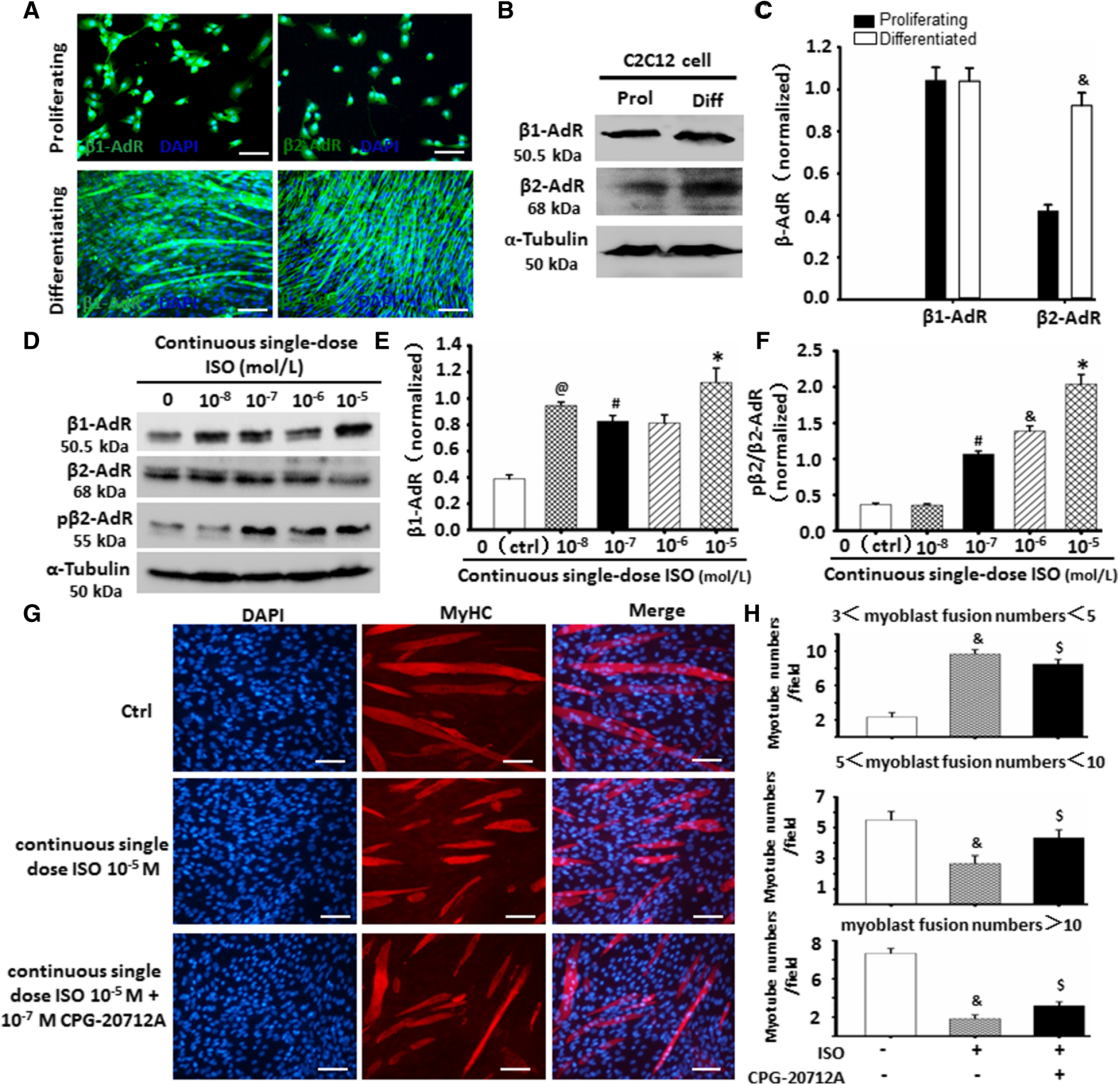
Continuous single-dose ISO delayed C2C12 cells differentiation and myoblast fusion through altering β-AdR. **a** The typical image of β1-AdR and β2-AdR expressions in proliferating or differentiated C2C12 cells as detected by immunofluorescent staining. Green color indicated corresponding AdR expression in C2C12 cells; blue color indicates DAPI-labeled nuclei. **b**-**c** The differentiated C2C12 cells compared with proliferating cells have shown the increased levels of β2-AdR as determined by Western blot, but no difference in β1-AdR. α-tubulin as the internal control. *P = 0.0019 vs. proliferating C2C12 cells; *n* = 3. **d**-**f** The traits of increased β1-AdR and pβ2-AdR in differentiated C2C12 cells with decreased β2-AdR when exposed to the stimulation of continuous single-dose ISO were determined by Western blot. α-tubulin as the internal control. ^@^*P* = 0.00014 vs. Ctrl; ^#^*P* = 0.0004 vs. 10^-8^ M ISO; ^&^*P* = 0.0002 vs. 10^-7^ M ISO; *P = 0.0016 vs. 10^-6^ M ISO; *n* = 3. **g** The typical image of myoblast fusion as determined by immunofluorescent staining of MyHC. Red color indicates MyHC; blue color indicates DAPI for nuclear labeling. **h** Continuous single-dose ISO decreased the myotube numbers of more than 5 myoblast fusion day 6 after C2C12 cells differentiation while increasing the myotube numbers of less than 5 myoblast fusion, the specific effects could be obviously abolished by β1-AdR inhibitor CPG-20712 (10^-7^ M). ^&^*P* = 0.0005 vs. Ctrl; ^$^*P* = 0.0012 vs. 10^-5^ M ISO; *n* = 3

In order to explore if continuous single-dose ISO-mediated C2C12 cell differentiation inhibition is involved in adrenergic receptors (AdRs), β1 and β2-AdRs in C2C12 cells were analyzed by using immunofluorescence staining. As shown in Fig. 4a, C2C12 cells expressed β1-AdR and β2-AdR. The differentiated C2C12 cells maintained a β1-AdR level similar to the proliferating cells. However, the differentiated C2C12 cells exhibited a markedly increased β2-AdR expression than the proliferating C2C12 cells (Fig. 4b, c), indicating that β2-AdR could involve in the process of C2C12 cell differentiation and myoblast fusion.

Since previous studies have shown that the reduced expression and sensitivity of β2-AdR in skeletal muscle involved in muscle atrophy during long-term SNS over-activation [8]. As shown in Fig. 4d–h, we found that continuous single ISO decreased β2-AdR levels while increasing phosphorylated β2-AdR (pβ2-AdR) in differentiated C2C12 cells, suggesting that the effects of continuous single ISO on delaying C2C12 cell differentiation and myoblast fusion could involve in inactivation of β2-AdR. In addition, continuous single ISO increased β1-AdR levels, and β1-AdR blocker CPG-20712 A could obviously reverse the inhibitory effects of continuous single ISO on C2C12 cell differentiation and myoblast fusion. Taken together, continuous ISO stimulation delayed C2C12 cell differentiation and myoblast fusion through altering β-AdR activities.

### The ratio of PKA RI/RII involved in the inhibitory effect of continuous ISO stimulation on C2C12 cell differentiation and myoblast fusion

**Fig. 5:**
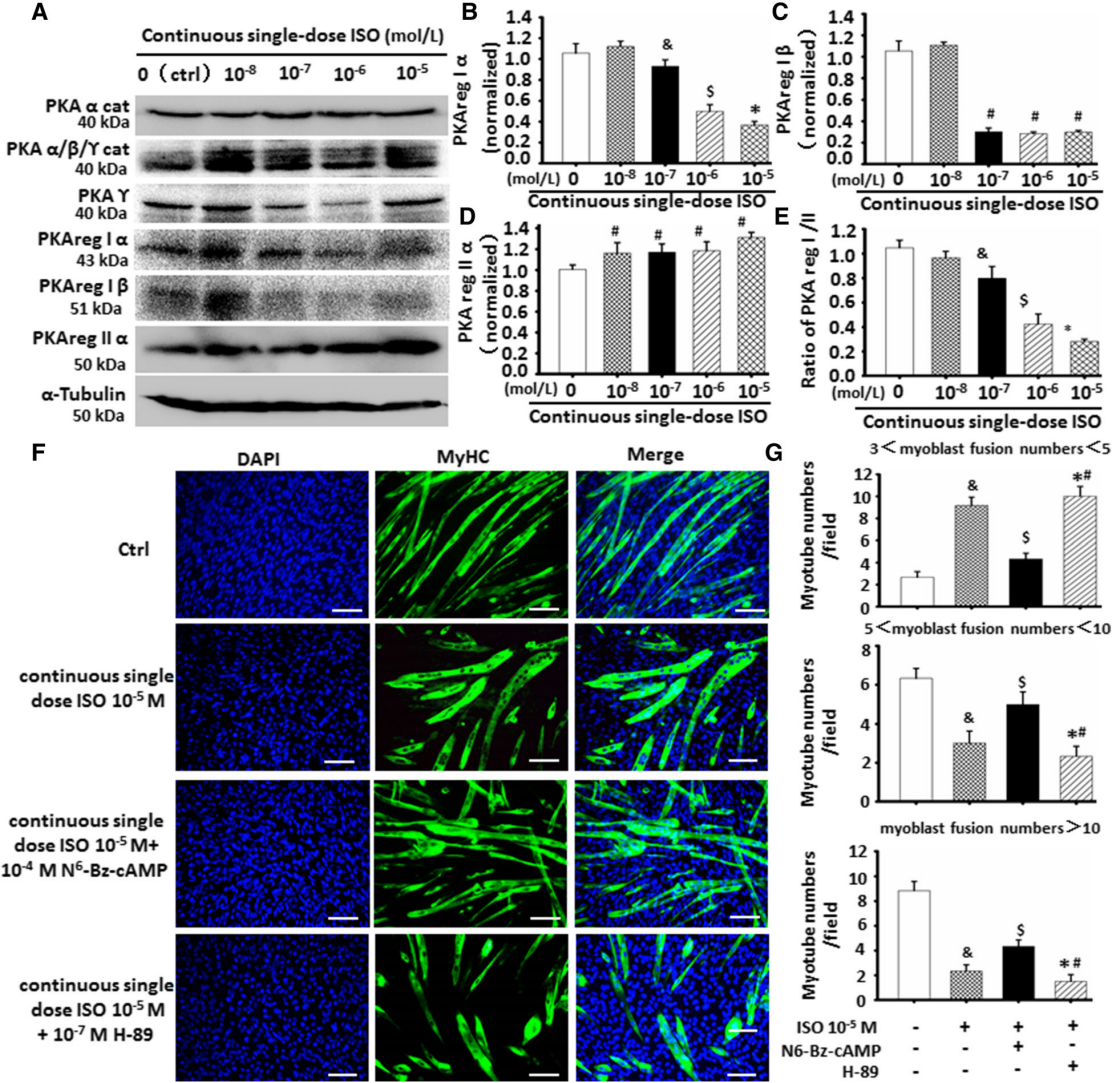
Continuous single-dose ISO delayed C2C12 cells differentiation and myoblast fusion through altering ratio of PKA RI/RII. **a** The expression of indicated proteins in continuous single-dose ISO-treated C2C12 cells were assessed by Western blot. α-tubulin as the internal control. **b**-**e** Continuous single-dose ISO obviously reduced levels of PKA RIα and PKA RIβ in C2C12 cells while increasing the levels of PKA RIIα as semi-quantitative analyses of PKA different subtypes band intensities. PKA RI/RII expressed the ratio of PKA RIα+PKA RIβ to PKA RIIα band intensities. ^#^*P* < 0.001 vs. another groups; ^&^*P* = 0.0014 vs. 10^-8^ M ISO; ^$^*P* = 0.0005 vs. 10*-7* M ISO; *P = 0.0019 vs. 10*-6* M ISO; *n* = 3. **f** The typical image of myoblast fusion of continuous single-dose ISO-treated C2C12 cells differentiation with or without PKA RI activator N^6^-Bz-cAMP (10^-4^ M) or PKA inhibitor H-89 (10^-7^ M) as determined by immunofluorescent staining of MyHC. Green color indicates MyHC; blue color indicates DAPI for nuclear labeling. **g** PKA RI activator partially abolished the inhibitory effects of continuous single-dose ISO on C2C12 cells differentiation while PKA inhibitor worsened its roles. ^&^*P* = 0.0004 vs. Ctrl; ^$^*P* = 0.0007 vs. 10^-5^ M ISO; *P < 0.05 vs. 10^-5^ M ISO; ^#^*P* = 0.0016 vs. N^6^-Bz-cAMP; *n* = 3

Since ISO exerts its effects by binding to β-AdR, generally resulting in activation of PKA [8], herein, we detected various subtypes of PKA by using western blot. As shown in Fig. 5a–e and Additional file 1: Figure S4, continuous single-dose ISO did not alter levels of PKA α cat, PKA α/β/ϒ cat, and PKA ϒ cat in C2C12 cells. However, PKA reg IIα showed an obvious increase in continuous single-dose ISO-treated C2C12 cells while decreasing PKA Riα and PKA RIβ, resulting in a decreased ratio of PKA RI/RII.

To explore if changes of ratio PKA RI/RII were involved in C2C12 cell differentiation and myoblast fusion, PKA RI agonist N^6^-Bz-cAMP and PKA inhibitor H-89 were used to confirm the specific effect. As shown in Fig. 5f and g, PKA RI agonist N^6^-Bz-cAMP could obviously reverse the inhibitory effects of continuous single-dose ISO on C2C12 cell differentiation and myoblast fusion. By contrast, PKA inhibitor H-89 further deteriorated the inhibitory effect (Fig. 5f, g). These results indicated that the traits of C2C12 cell differentiation and myoblast fusion following the stimulation of continuous single-dose ISO could be associated with abnormal changes of ratio of PKA RI/RII.

### The involvement of ERK1/2 in inactivating β2-AdR by continuous single ISO

**Fig. 6:**
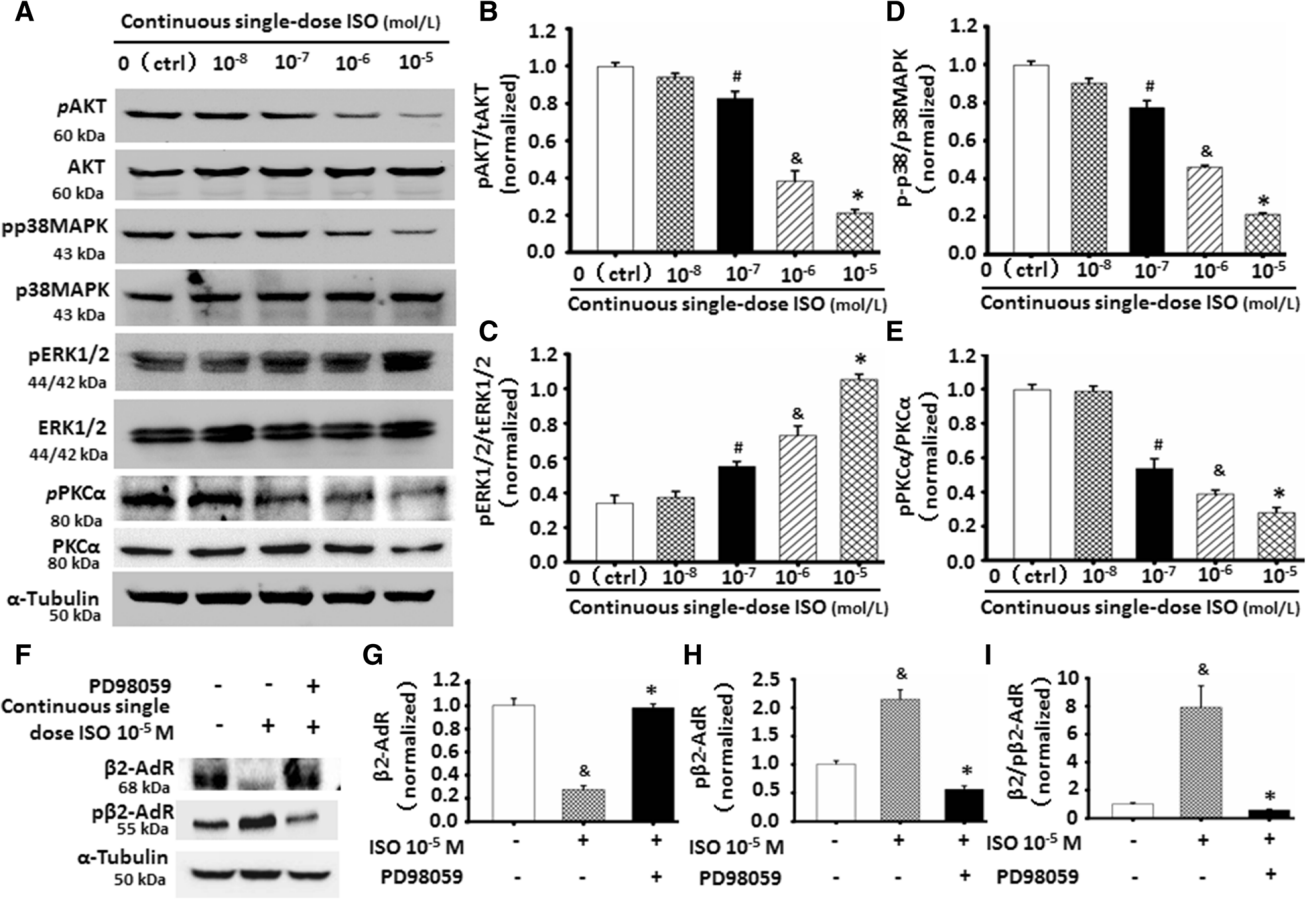
Inactivation of β2-AdR by continuous single-dose ISO involved in ERK1/2 signaling. **a** The expression of indicated proteins were assessed by western blot. α-tubulin as the internal control. **b**-**e** The increased pERK1/2 and decreased pAKT and p38MAPK in continuous single-dose ISO-treated C2C12 cells were shown by semi-quantitative analyses of the indicated proteins in Fig.6a by normalizing to the corresponding total protein. ^#^*P* = 0.0101 vs. Ctrl or 10^-8^ M ISO; ^&^*P* = 0.0003 vs. 10^-7^ M ISO; *P = 0.0068 vs. 10^-6^ M ISO; *n* = 3. **f**-**i** Continuous single-dose ISO decreased β2-AdR levels while increasing pβ2-AdR levels, the specific effect could be almost completely abolished by PD98059 (5х10^-5^ M) as determined by western blot. ^&^*P* = 0.0014 vs. Ctrl; *P = 0.0008 vs. 10^-5^ M ISO; *n* = 3

Previous studies have shown that activated PKC, ERK1/2, and p38MAPK signaling following repeated or long-term administration of β-AdR agonist including ISO are involved in the reduced expression and sensitivity of β2-AdR, apart from PKA [20–23]. As shown in Fig. 6a, continuous stimulation by ISO did not alter the total PKC, AKT, ERK1/2, and p38MAPK levels in the C2C12 cells. However, it inhibited the activation or phosphorylation of PKC, AKT, and p38MAPK (Fig. 6a–e) while increasing the activation of ERK1/2 (Fig. 6a, c) in a dosage-dependent manner. Combining the decreased levels of phosphorylation of β2-AdR (pβ2-AdR) following the stimulation of continuous single-dose ISO (Fig. 4d–f), these results indicated that activated ERK1/2 signaling could be involved in phosphorylation of β2-AdR. Subsequently, ERK1/2 inhibitor PD98059 were used to further confirm if ERK1/2 was involved in the increase of pβ2-AdR levels in the ISO-treated C2C12 cell, the result showed that the special role of continuous single-dose ISO in increasing the levels of pβ2-AdRcould be markedly abolished by PD98059 (Fig. 6f–h). Therefore, these data indicated that continuous single ISO-mediated inactivation of β2-AdR was involved in ERK1/2 signaling.

### ERK1/2-mediated inactivation of β2-AdR by ISO involved in C2C12 cell differentiation and myoblast fusion

**Fig. 7:**
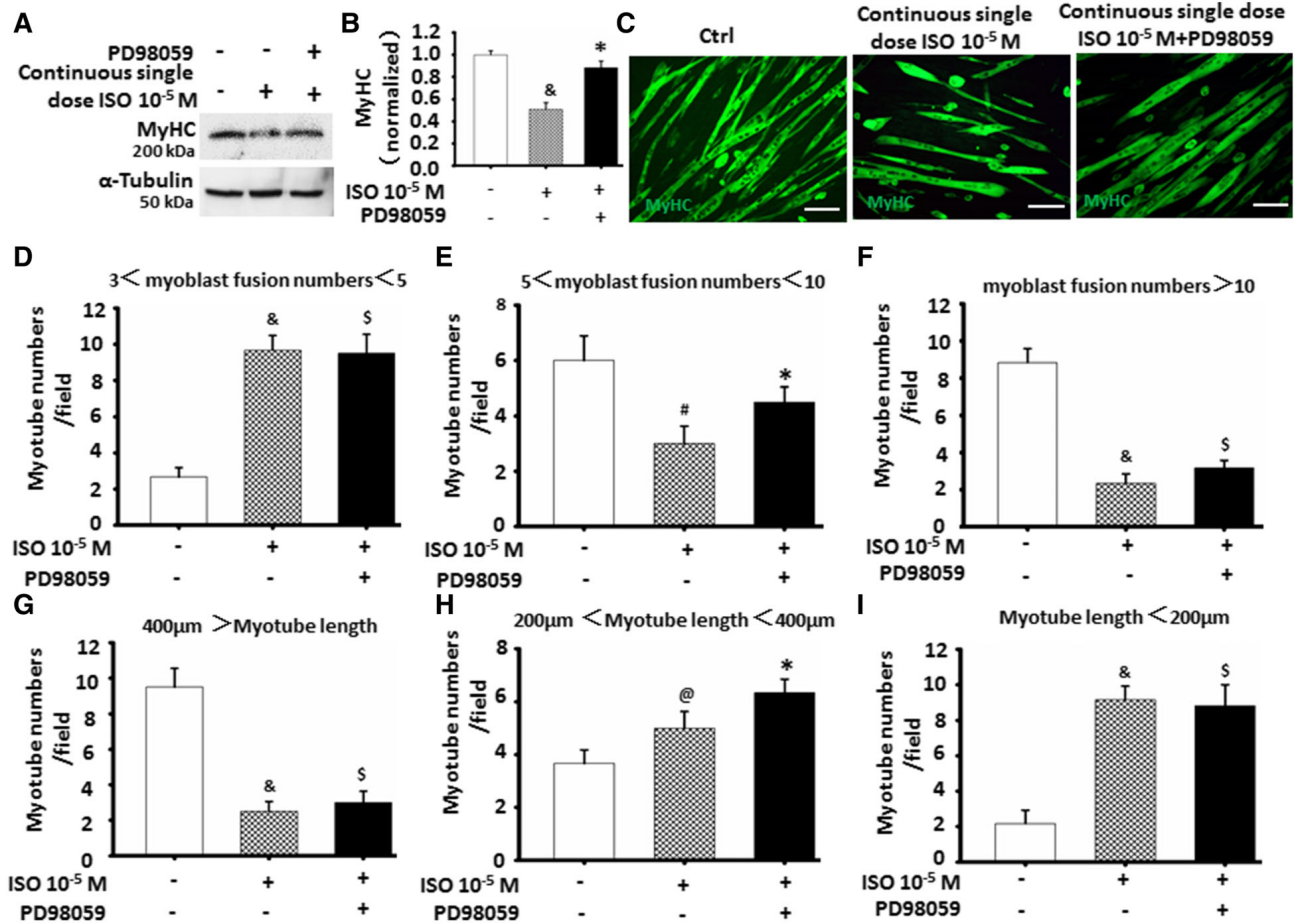
ERK1/2-mediated inactivation of β2-AdR involved in C2C12 cells differentiation and myoblast fusion. **a**-**b** Continuous single-dose ISO decreased MyHC expressions, the specific effect could be partially abolished by PD98059 as determined by western blot. ^&^*P* = 0.0016 vs. Ctrl; *P = 0.0011 vs. 10^-5^ M ISO; *n* = 3. **c** The typical image of myoblast fusion as determined by immunofluorescent staining of MyHC. Green color indicates MyHC; blue color indicates DAPI for nuclear labeling. **d**-**f** PD98059 obviously abrogated the effects of continuous single-dose ISO on the numbers of MyHC positive myotube derived from C2C12 cells differentiation. ^&^*P* = 0.002 vs. Ctrl; ^$^*P* < 0.05 vs. 10^-5^ M ISO; *P = 0.0014 vs. 10^-5^ M ISO; ^@^*P* = 0.041 vs. 10^-5^ M ISO; *n* = 6. **g**-**i** PD98059 partially abolished the effects of ISO on myotube numbers with different length derived from C2C12 cells differentiation. ^&^*P* = 0.0000 vs. Ctrl; ^@^*P* = 0.0025 vs. Ctrl; *P = 0.009 vs. 10^-5^ M ISO; ^$^*P* < 0.05 vs. 10^-5^ M ISO. *n* = 6

To further determine the role of ERK1/2-mediated inactivation of β2-AdR by continuous single-dose ISO in attenuating C2C12 cell differentiation and myoblast fusion, we firstly observed the expression of MyHC. As shown in Fig. 7a and b, ERK1/2 inhibitor PD98059 could remarkably abolish the inhibitory role in MyHC expressions. Secondly, immunofluorescence staining of MyHC was used to evaluate C2C12 cell differentiation and myoblast fusion (Fig. 7c). Finally, we quantificationally analyzed the changes of myotube morphology. As shown in Fig. 7d–i, PD98059 obviously increased the numbers of myotube with 5 to 10 myoblast fusions (Fig. 7d), coupling with the increased numbers of myotube with a length of 200 to 400 μm (Fig. 7h). Combining the results of Fig. 6, ERK1/2 signaling might be involved in the inhibitory effects of continuous single-dose ISO on C2C12 cell differentiation and myotube formation through inactivating β2-AdR.

### ERK1/2 involved in C2C12 cell differentiation and muscle fiber types through FoxO1 signaling

**Fig. 8:**
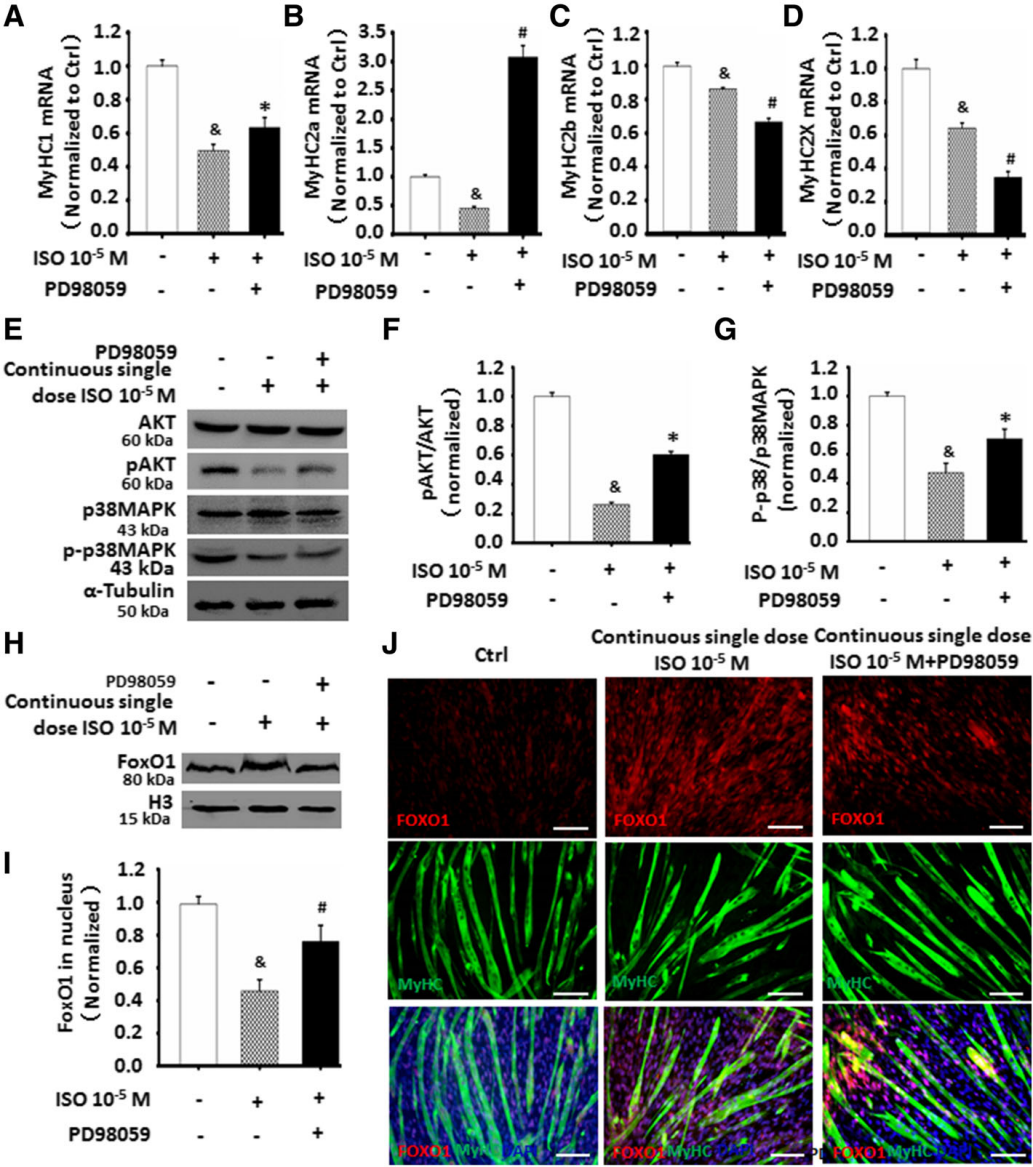
ERK1/2 involved in C2C12 cells differentiation and muscle fiber types through FoxO1 signaling. **a**-**d** ERK1/2 inhibitor PD98059 partially abrogated the role of continuous single-dose ISO in repressing the expression of MyHC1 and MyHC2a as detected by Real-time PCR, in addition to further reducing the mRNA levels of MyHC2b and MyHC2x. ^&^*P* = 0.0000 vs. Ctrl; *P = 0.0292 vs. 10^-5^ M ISO; ^#^*P* = 0.0004 vs. Ctrl; *n* = 6. **e**-**g** PD98059 in part recovered the decreased levels of *p*AKT, and *p*-p38MAPK in ISO-treated C2C12 cells as determined by Western blot and band intensities. α-tubulin as the internal control. ^&^*P* = 0.0004 vs. Ctrl; *P = 0.0122 vs. 10^-5^ M ISO; *n* = 3. **h**-**i** ERK1/2 inhibitor PD98059 partially decreased the levels of nuclear FOXO1 in continuous single-dose ISO-treated C2C12 cells as analyzed by Western blot band intensities. ^&^*P* = 0.0004 vs. Ctrl; ^#^*P* = 0.0124 vs. 10^-5^ M ISO; *n* = 3. **j** ERK1/2 inhibitor PD98059 partially reduced nuclear translocation of FOXO1 in differentiated C2C12 cells induced by ISO as detected by immunofluorescent staining. Red: FOXO1; green: MyHC; blue: DAPI-labeled nuclei

Recent studies have shown that AKT, ERK1/2, and p38MAPK signaling are involved in the regulation of C2C12 cell differentiation and myoblast fusion ([24–26], and). In consistent with the results of Figs. 6 and 7, using real-time PCR to further evaluate the changes of muscle fiber types in continuous single-dose ISO-treated C2C12 cell, especially in coexistence of ISO with PD98059, we found that PD98059 increased the expression of MyHC1 and MyHC2a while decreasing the expression of MyHC2b and MyHC2X (Fig. 8a–d) as compared with the ISO-treated group, indicating ERK/1/2 was important for the formation of myotube.

FOXO1, a downstream target of AKT and p38 MAPK [27, 28], plays a crucial role in muscle satellite cell differentiation and myoblast fusion, especially in I type fiber differentiation [29, 30]. In line with the decreased levels of pAKT and p-p38 MAPK in Fig. 6, as shown in Additional file 1: Figure S3, continuous stimulation by ISO gradually decreased the levels of FOXO1phosphorylation in C2C12 cell cytoplasm, leading to the increase of nuclear FOXO1 levels (Fig. 8e–i), indicating that highly activated FOXO1 is involved in the inhibition of myotube formation. Accompanied by the partial recovery of pAKT and p-p38 MAPK levels in the ISO-treated C2C12 cells, PD98059 decreased the levels of nuclear FOXO1 (Fig. 8h, i). Meanwhile, a significantly more nuclear translocation of FOXO1 was observed in the cells with continuous ISO stimulation as compared to the normally differentiated cells as shown by the double immunostaining of MyHC and FOXO1 (Fig. 8j). Furthermore, PD98059 partially recovered the nuclear translocation of FOXO1, in similar to normal state of differentiated C2C12 cells. Taken together, these data suggested that FOXO1 activation by ISO correlated with C2C12 cell differentiation and myoblast fusion, which was associated with ERK1/2 signaling.

## Discussion

Due to hyperexcitability of SNS, the damage triggered by the increased levels of NE and E often attributes to the activation of β-AdR [7]. Since NE and E can bind to both the α-AdR and β-AdR, causing difficulty to analyze the unique role of each subtype of AdRs [2], a more stable β-AdR-binding isoproterenol (ISO) has been used to mimic the effect of β-AdR activation. In previous studies, transient stimulation with a single high dose of ISO (10^−4^ M) is used to induce skeletal muscle atrophy [31, 32]. In this study, we established a clinically relevant chronic activation of β-AdR, i.e., continuous stimulation with ISO, to observe its effect on C2C12 cell differentiation and myoblast fusion. Our studies made three novel observations. Firstly, activation of β1-AdR and inactivation of β2-AdR by continuous ISO stimulation contributed to the decreased ratio of PKA RI/RII, causing a greater inhibition of C2C12 cell differentiation and myoblast fusion. Secondly, continuous ISO stimulation desensitized β2-AdR by activating ERK1/2 signaling. And lastly, continuous stimulation of ISO inhibited C2C12 cell differentiation and myoblast fusion, leading to the decrease of type I and type II myotube formation, at least in part, by activating the ERK1/2-FOXO1 signaling.

Traditionally, the stimulated AdRs have been thought to couple with the PKA activation mediated by G-protein/AC. Stimulation of this pathway has been linked to myoblast differentiation and myotube formation, attributing to the activation of PKA RI during physiological condition [33]. In continuous ISO stimulation system, as a typical simulation model in vitro for excessive SNS-associated pathological state in vivo, we found that it decreased the levels of both PKA RIα and RIβ while increasing PKA RIIα levels, resulting in delaying C2C12 cell differentiation and reducing myotube formation. Further evidences have shown that continuous ISO stimulation increased β1-AdR expression while decreasing β2-AdR levels linked to the levels of pβ2-AdR, which were associated with activation of ERK1/2 signaling. Our studies have demonstrated β1-AdR antagonist CPG-20712A partially abolish the inhibitory effect of ISO on C2C12 differentiation and myoblast fusion. Recent reports have shown that β2-AdR agonists including clenbuterol or fenoterol increased skeletal muscle mass [34, 35]. And ERK1/2 inhibitor PD98059 recovered the levels of β2-AdR, leading to the recovery of C2C12 differentiation and myoblast fusion, consistent with its role in restoring the differentiation potential of Ras-transformed C2C12 myoblasts [36]. Therefore, the changes of ERK1/2-mediated β-AdR activities induced by continuous ISO stimulation could cause the alteration of ratio of PKA RI/RII, triggering the abnormal myoblast differentiation and myotube fusion.

As a result of phosphorylation, inactivation of β2-AdR was frequently controlled by PKA, PKC, p38MAPK, ERK1/2, and G-protein-coupled receptor kinases (GRKs) [37–39]. In the present study, accompanied by increased pβ2-AdR levels, β2-AdR levels were remarkably decreased in C2C12 cells following continuous ISO stimulation, attributing to the activation of ERK1/2. Indeed, previous studies have shown that pERK1/2 could accelerate β2-AdR desensitization through the process of catalyzing the hydrolysis of cAMP into AMP by inducing the expression of phosphodiesterase 4 (PDE4) mediated by the activated CREB (cAMP response element binding protein), apart from triggering GRK4 to phosphorylate β2-AdR [40–42]. Of interest, continuous single-dose ISO-treated C2C12 cells showed the unique characteristics of active separation of both β1-AdR and β2-AdR, indicating that other unknown mechanisms could involve this particular phenomenon, which could be further explored in the future.

Published data have shown that FOXO1 play a crucial role in myotube formation, especially type I myotube formation [29, 30]. FOXO1 activities are frequently regulated by PI3K/AKT and p38MAPK pathway during C2C12 cell differentiation [24–26]. Herein, we found that continuous ISO stimulation decreased the levels of pAKT while increasing the pERK1/2 levels, resulting in more FOXO1 levels. Conversely, ERK1/2 inhibitor PD98059, at least in part, through recovering the levels of pAKT and p-p38MAPK, reduced nuclear translocation of FOXO1 and the levels of nuclei FOXO1in C2C12 cells, contributing to the partial recovery of C2C12 cell differentiation and myotube formation, mainly in type II myotube formation. Of interest, a constitutively active FOXO1 mutation at the AKT phosphorylation site inhibits C2C12 cell differentiation and type I myotube formation [29, 30]. Since continuous ISO stimulation inhibits the formation of both type I and II myotubes, factors other than FOXO1 may also be involved in C2C12 cell differentiation and fusion, which could be studied in the future.

One limitation of this study is the lack of in vivo evidence that the excessive SNS-muscular dystrophy is involved in dysfunction of skeletal muscle satellite cells and corresponding abnormal signal pathways. In the future, we could use Duchenne and Becker muscular dystrophy and/or long-term sympathetic nerve hyperactivity models to analyze muscle satellite cell function and signaling pathways involved, and test if continuous stimulation with ISO inhibits satellite cell and myoblast fusion. These in vivo studies may ultimately provide strategies to treat MD through correcting the autonomic imbalance or controlling the signaling involved.

## Conclusion

Continuous stimulation with ISO appears to be a valuable model for studying the excessive SNS-associated muscle satellite cell function. Our results reveal a novel mechanism underlying the ISO inhibition in C2C12 cell differentiation and myoblast fusion, i.e., ISO binds to AdR, which alters the ratio of PKA RI/RII and activates ERK1/2 while inhibiting PKC, AKT, and p38MAPK signaling, leading to over-activation of FOXO1, thus inhibiting C2C12 cell differentiation and myotube formation.

## Additional file

Additional file 1: **Figure S1.** Continuous single-dose ISO obviously inhibited C2C12 cell differentiation and myoblast fusion than single-dose ISO. **Figure S2.** Continuous ISO stimulation altered myoblast fusion competence of myotube during C2C12 cell differentiation. **Figure S3.** Continuous single ISO involved in C2C12 cell differentiation and muscle fiber types through FoxO1 signaling. **Figure S4.** Continuous single ISO did not alter levels of PKA α cat, PKA α/β/ϒ cat, PKA ϒ cat in C2C12 cell differentiation. (DOCX 519 kb)

## Abbreviations

AC: Adenylate cyclase
AdR: Adrenergic receptor
AKT: Protein Kinase B
DAPI: 4′,6-Diamidino-2-Pheny lindole
DCM: Dilated cardiomyopathy
DMEM: Dulbecco’s modified Eagle’s medium
E: Epinephrine
ERK1/2: Extracellular signal-regulated kinase1/2
FBS: Fetal bovine serum
FOXO1: Forkhead box protein O1
HF: Heart failure
HRP: Horseradish peroxidase
HS: Horse serum
IF: Immunofluorescence;
IGF-1: Insulin-like growth factor
ISO: Isoprenaline
M: Mol/L
MAPK: Mitogen-activated protein kinase
MD: Muscular dystrophy
MuRF1: Muscle-specific RING-finger protein 1
MyHC: Myosin heavy chain
MyHC1: Myosin heavy chain 1
MyHC2a: Myosin heavy chain 2a
MyHC2b: Myosin heavy chain 2b
MyHC2x: Myosin heavy chain 2x
MyoD: Myogenic differentiation 1
MyoG: Myogenin
NE: Norepinephrine
PBS: Phosphate buffered saline
PKA RIIα: PKA regulatory subunit IIα
PKA RIIβ: PKA regulatory subunit IIβ
PKA RIα: PKA regulatory subunit Iα
PKA RIβ: PKA regulatory subunit Iβ
PKA α/β/γ cat: PKA α/β/γ catalytic subunits
PKA: Protein kinase A
PKAα cat: PKA alpha catalytic subunit
PKAβ cat: PKA beta catalytic subunit
PKC: Protein Kinase C
PVDF: Polyvinylidene fluoride
qPCR: Quantitative polymerase chain reaction
SNS: Sympathetic nerve system
