# Supplementary material for: Continuous exposure of isoprenaline inhibits myoblast differentiation and fusion through PKA/ERK1/2-FOXO1 signaling pathway
Source: Stem Cell Res Ther. 2019 Feb 28;10:70. doi: 10.1186/s13287-019-1160-x (PMC6394105; doi:10.1186/s13287-019-1160-x)
Supplement: Supplementary file 1 — Figure S1. Continuous single-dose ISO obviously inhibited C2C12 cell differentiation and myoblast fusion than single-dose ISO. Figure S2. Continuous ISO stimulation altered myoblast fusion competence of myotube during C2C12 cell differentiation. Figure S3. Continuous single ISO involved in C2C12 cell differentiation and muscle fiber types through FoxO1 signaling. Figure S4. Continuous single ISO did not alter levels of PKA α cat, PKA α/β/ϒ cat, PKA ϒ cat in C2C12 cell differentiation. (DOCX 519 kb) [file 13287_2019_1160_MOESM1_ESM.docx]

**Additional file 1**

**Continuous exposure of isoprenaline inhibits myoblast differentiation and fusion through** **PKA/ERK1/2-FOXO1 signaling pathway**

Shao-juan Chen^1, 2, 3#^, Jing Yue^1, 3#^, Jing-Xuan Zhang^3,5^, Miao Jiang^1^, Tu-qiang Hu^4^, Wei-dong Leng^2^, Li Xiang ^1, 3^, Xin-yuan Li^1^, Lei Zhang^1,5^, Fei Zheng^1^, Ye Yuan^1^, Lin-yun Guo^1,5^, Ya-mu Pan^1^, Yu-wen Yan^1^, Jia-ningWang^1,5^, Shi-You Chen^6^, Jun-mingTang^1,3,5*^

^1^Department of Cardiology, and Institute of Clinical Medicine, Renmin Hospital, Hubei University of Medicine, Hubei 442000, PR China

^2^Department of Stomatology, Taihe Hospital, Hubei University of Medicine, Shiyan, Hubei 442000, PR China

^3^Department of Physiology, School of Basic Medical Sciences, Hubei University of Medicine, Shiyan, Hubei 442000, PR China

^4^Department of Stomatology, Renmin Hospital, Hubei University of Medicine, Shiyan, Hubei 442000, PR China

^5^Institute of biomedicine and Key Lab of Human Embryonic Stem Cell of Hubei Province, Hubei University of Medicine, Hubei 442000, China

^6^Department of Physiology & Pharmacology, The University of Georgia, Athens, GA30602, USA

^#^Co-first author; *Corresponding Author: Jun-ming Tang

Institute of Clinical Medicine, Renmin Hospital, Hubei University of Medicine, Shiyan, Hubei 442000, China

Phone: 86-719-8637171, Email: tangjm416@163.com (Tang)

**Additional file 1: Figure S1**

**
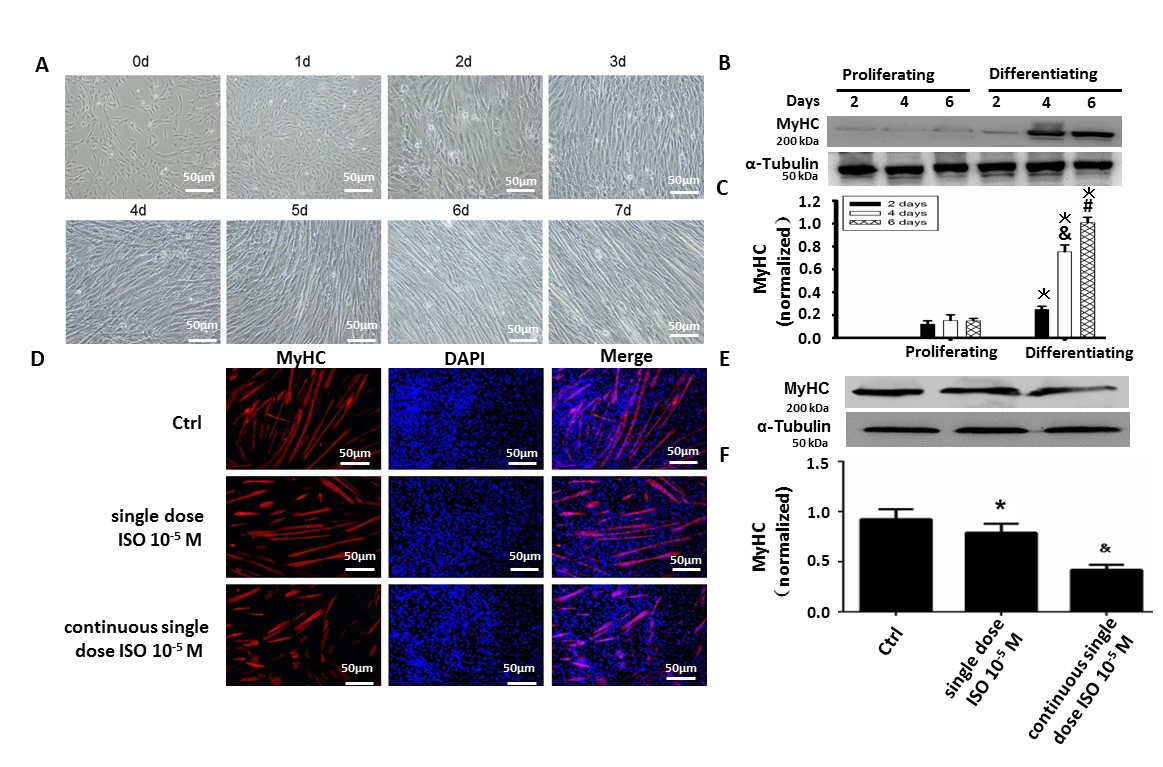
**

**Figure S1. Continuous single-dose ISO obviously inhibited C2C12 cells differentiation and myoblast fusion than single-dose ISO**

**(A)** Cell morphological alteration during C2C12 cell differentiation. (B) MyHC expression was detected by Western blot. (C) Semi-quantitative analyses of MyHC level shown in A by normalizing to the internal control α-tubulin. ^*^*P=*0.0022 vs. proliferating C2C12 cells; ^&^*P=*0.0002 vs. 2 days; ^#^*P=*0.0042 vs. 4 days; n=3. (D) Continuous stimulation with ISO caused greater inhibition of C2C12 cell differentiation compared to the transient stimulation (single dose), as assessed by immunofluorescent cytochemical staining of MyHC. Red color indicated MyHC expressions in differentiated C2C12 cells; blue color indicates DAPI-labeled nuclei. (E) MyHC expression was determined by Western blot. (F) Semi-quantitative analyses of MyHC in Figure S1 by normalizing to the internal control α-tubulin. *P=0.019 vs. Ctrl; ^&^P=0.0004 vs. single dose; n=6.

**Additional file 1: Figure S2**


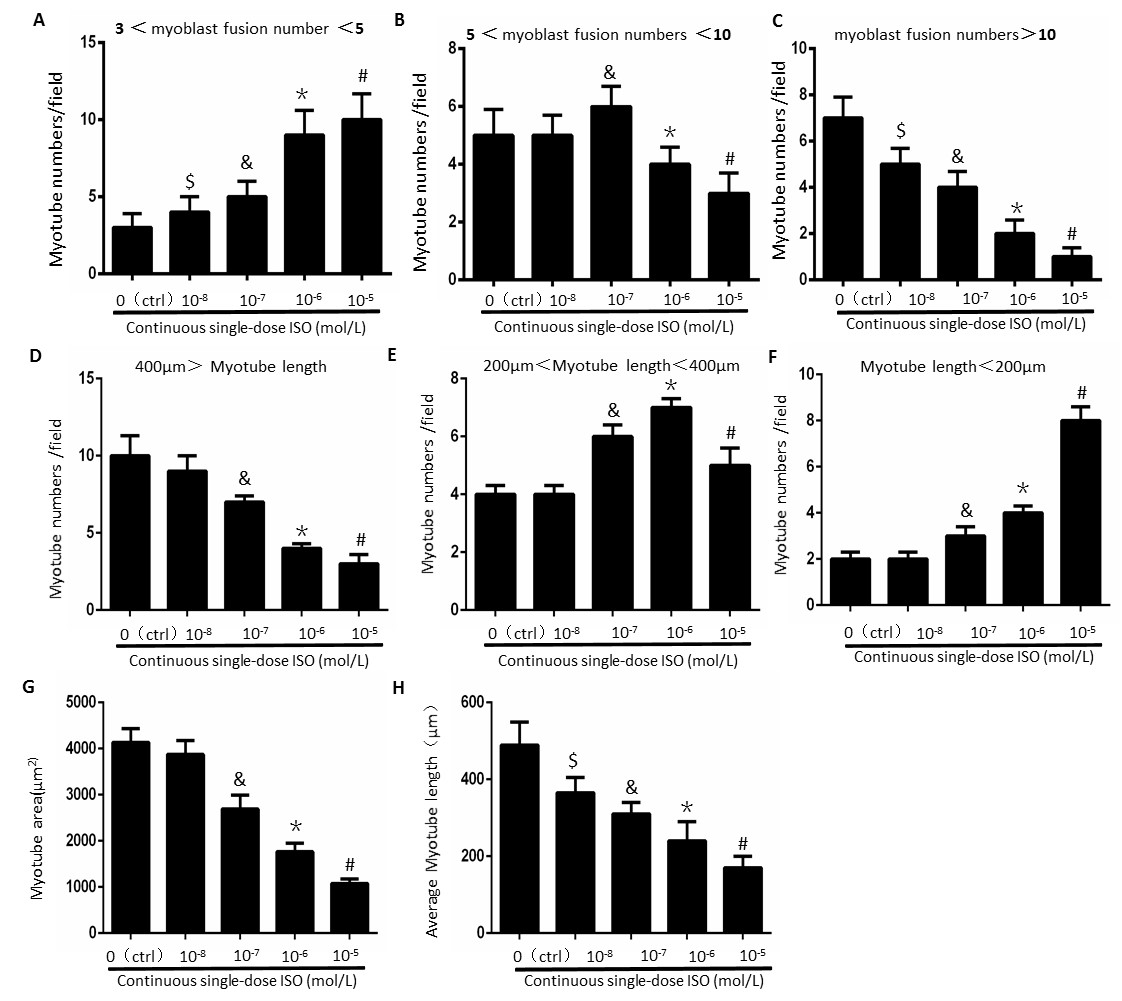


**Figure S2. Continuous ISO stimulation altered myoblast fusion competence of myotube during C2C12 cell differentiation.** (A-C) Dose-dependent effects of ISO on myotube numbers with indicated myoblast fusion numbers/field. (D-F) Dose-dependent effects of ISO on myotube numbers with indicated myotube length. (G-H) Dose-dependent effects of ISO on myotube area and average myotube length in differentiated C2C12 cells. ^$^*P=*0.0235 vs. Ctrl; ^&^*P=*0.0194 vs. 10^-8^M ISO; ^*^*P=*0.0206 vs. 10^-7^ M ISO; ^#^*P=*0.0136 vs. 10^-6^ M ISO; n=6.

**Additional file 1: Figure S3**


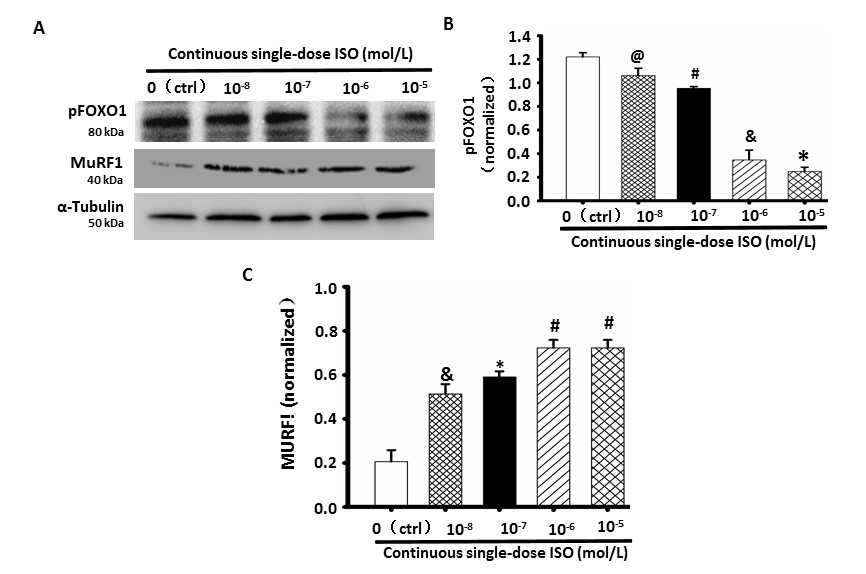


**Figure S3. Continuous single ISO involved in C2C12 cells differentiation and muscle fiber types through FoxO1 signaling**

(A) pFOXO1 and MuRF1 expression were detected by Western blot. (B) Semi-quantitative analysis of pFOXO1 levels in figure S3A by normalizing to the internal control α-tubulin. ^@^*P=*0.0015 vs. Ctrl; ^#^*P=*0.0640 vs.10^-8^ M ISO; ^&^*P=*0.0062 vs. 10^-7^ M ISO; ^*^*P=*0.0062 vs. 10^-7^ M ISO; n=3. (C) Semi-quantitative analysis of MuRF1 levels in figure S3A by normalizing to the internal control α-tubulin. ^&^*P=*0.0015 vs. Ctrl; ^*^*P=*0.0640 vs.10^-8^ M ISO; ^#^*P=*0.0062 vs. 10^-7^ M ISO; n=3.

**Additional file 1: Figure S4**


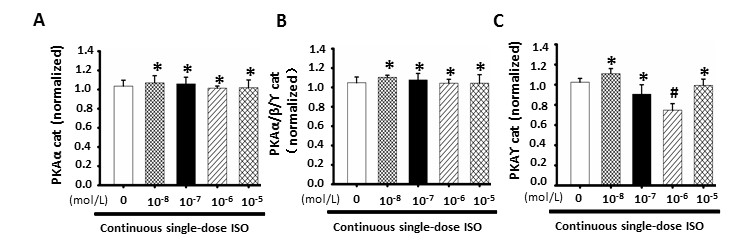


**Figure S4.** **Continuous single ISO did not alter levels of PKA α cat, PKA α/β/ϒ cat, PKA ϒ cat in C2C12 cells differentiation.** Semi-quantitative analysis of PKA α cat, PKA α/β/ϒ cat, PKA ϒ cat by normalizing to the internal control α-tubulin. ^*^*P＞ 0.05* vs. other groups; ^#^*P＜0.05* vs. other groups; n=3.
